# Association of Diabetes Severity and Mortality with Lung Squamous Cell Carcinoma

**DOI:** 10.3390/cancers14102553

**Published:** 2022-05-22

**Authors:** Chih-Hsiung Su, Wan-Ming Chen, Mingchih Chen, Ben-Chang Shia, Szu-Yuan Wu

**Affiliations:** 1 Department of Accounting Information, Chihlee University of Technology, Taipei 22050, Taiwan; suhsiung@gmail.com; 2 Graduate Institute of Business Administration, College of Management, Fu Jen Catholic University, Taipei 24205, Taiwan; daisywanmingchen@gmail.com (W.-M.C.); 081438@mail.fju.edu.tw (M.C.); 025674@mail.fju.edu.tw (B.-C.S.); 3 Artificial Intelligence Development Center, Fu Jen Catholic University, Taipei 24205, Taiwan; 4 Department of Food Nutrition and Health Biotechnology, College of Medical and Health Science, Asia University, Taichung 41354, Taiwan; 5 Division of Radiation Oncology, Lo-Hsu Medical Foundation, Lotung Poh-Ai Hospital, Yilan 265501, Taiwan; 6 Big Data Center, Lo-Hsu Medical Foundation, Lotung Poh-Ai Hospital, Yilan 265501, Taiwan; 7 Department of Healthcare Administration, College of Medical and Health Science, Asia University, Taichung 41354, Taiwan; 8 Cancer Center, Lo-Hsu Medical Foundation, Lotung Poh-Ai Hospital, Yilan 265501, Taiwan; 9 Centers for Regional Anesthesia and Pain Medicine, Taipei Municipal Wan Fang Hospital, Taipei Medical University, Taipei 110301, Taiwan; 10 Department of Management, College of Management, Fo Guang University, Yilan 26247, Taiwan

**Keywords:** severity of diabetes, lung cancer, survival, squamous cell carcinoma, propensity scores matching

## Abstract

**Simple Summary:**

The survival impact of diabetes severity on lung cancer survival remains unclear. We performed head-to-head propensity score matching to estimate the survival impact of various adapted diabetes complications severity index (aDCSI) scores in patients with both diabetes and lung squamous cell carcinoma (SqCLC). The results indicated that diabetes severity (aDCSI ≥ 2) is an independent prognostic factor for the overall survival of patients with both diabetes and lung SqCLC who receive standard treatments. Prevention of diabetes progression is necessary for patients with diabetes; it affects not only diabetes control but also improves survival for patients with lung SqCLC.

**Abstract:**

Purpose: The survival impact of diabetes severity on lung cancer remains unclear. We performed head-to-head propensity score matching to estimate the survival impact of various adapted diabetes complications severity index (aDCSI) scores in patients with both diabetes and lung squamous cell carcinoma (SqCLC). Patients and Methods: We enrolled patients with both diabetes and lung SqCLC and categorized them into the mild (aDCSI = 0–1) and moderate-to-severe (aDCSI ≥ 2) diabetes groups. The patients in both groups were matched at a 1:1 ratio. Results: the matching process yielded a final cohort of 5742 patients with both diabetes and lung SqCLC (2871 patients in the mild diabetes group and 2871 patients in the moderate-to-severe diabetes groups) who were eligible for further analysis. A multivariate Cox regression analysis revealed that the adjusted hazard ratio (aHR; 95% confidence interval) of all-cause death for the mild diabetes group relative to the moderate-to-severe diabetes group was 1.17 (1.08–1.28; *p* = 0.0005). Conclusion: severe diabetes (aDCSI ≥ 2) is an independent prognostic factor for OS among patients with both diabetes and lung SqCLC who receive standard treatments. Preventing diabetes progression is necessary for patients with diabetes because it not only supports diabetes control but also improves survival for patients with lung SqCLC.

## 1. Introduction

Having a high blood sugar level can damage various organ systems, especially the cardiovascular and nervous systems [1,2]. Diabetes can lead to cardiovascular disease, nephropathy, retinopathy, peripheral vascular disease, stroke, neuropathy, and metabolic complications [2]. Recent studies have verified that diabetes is linked to dementia, hearing loss, and specific forms of cancer [3,4,5]. Diabetes is associated with increased incidence and mortality in numerous types of cancer [6,7,8], and it may influence cancer progression and outcomes [9]. In addition, diabetes mellitus is a risk factor for all-cause death in patients with cancer [9].

Lung cancer is the leading and second leading cause of cancer death worldwide in men and women, respectively [10]. Diabetes may increase the risk of lung cancer, and it is associated with poor overall survival (OS) among women with lung cancer [11]. The potential mechanisms of poor survival in patients with both diabetes and lung cancer can be attributed to hyperinsulinemia and hyperglycemia with poor diabetes control and the occurrence of diabetic complications, which contribute to lung cancer progression and poorer survival outcomes among such patients [11]. Nevertheless, no study has produced clinical evidence to demonstrate the association of diabetes severity with the survival outcomes of lung cancer.

The adapted Diabetes Complications Severity Index (aDCSI) is used to assess diabetes severity for the purpose of predicting mortality rates, hospitalization rates, and medical costs [12]. However, the survival impact of diabetes severity on lung cancer remains unclear. In the current study, we performed head-to-head propensity score matching (PSM) to estimate the survival impact of various aDCSI scores among patients with both diabetes and lung squamous cell carcinoma (SqCLC). We focused on lung SqCLC because of the lack of heterogeneous treatments and the absence of evidence regarding the contribution of specific driver gene mutations to improved or poorer survival. Information on the association of diabetes severity with survival outcomes for lung SqCLC can serve as a valuable reference for health authorities, particularly in terms of the formulation of health policies aimed at preventing diabetes progression and improving survival in patients with lung SqCLC. Our findings help to clarify why patients who have similar clinical lung cancer stages and receive similar treatments exhibit different survival outcomes.

## 2. Patients and Methods

### 2.1. Data Sources and Study Cohort

From the Taiwan Cancer Registry database, we identified patients with mild diabetes or moderate-to-severe diabetes who received a diagnosis of lung SqCLC between 1 January 2008 and 31 December 2018. Patients were classified as having mild diabetes and moderate-to-severe diabetes if they had aDCSI scores of 0–1 and ≥2, respectively, during the 3 months following the date of a lung cancer diagnosis [2,12]. The aDCSI is a good measure of diabetes severity [13]. The complications severity index was categorized into 2 or 3 levels (no abnormality = 0, some abnormality = 1, and severe abnormality = 2), depending on the presence and severity of the complication. If no abnormalities were present, the patient received no score for that complication. If a patient had any complication classified as some abnormality, a 1 was added to the DCSI. If patients had any complication classified as severe abnormality, a 2 was added (Table S1) [2]. Due to severe abnormality, a 2 was added [2], we compared the OS between SqCLC patients with aDCSI scores of 0–1 and those with aDCSI scores of ≥2. The index date was the date of lung cancer diagnosis, and the follow-up duration was from the index date to 31 December 2019. The study protocols were reviewed and approved by the Institutional Review Board of Tzu-Chi Medical Foundation (IRB109-015-B). Furthermore, we used data from the cancer registry database of the Collaboration Center of Health Information Application, which archives cancer-related information regarding pathological types, cancer stages, and treatments [14,15,16,17]. The vital status and the cause of death of each patient were verified.

### 2.2. Patient Selection

#### Inclusion and Exclusion Criteria

Patients with diabetes were included if they were diagnosed as having lung SqCLC on the basis of pathological reports; were aged ≥20 years; and had lung SqCLC (stages I–IIIC) without metastasis as defined by the American Joint Committee on Cancer (AJCC, 8th edition). Patients with diabetes were excluded if they had a history of cancer before receiving their diagnosis of lung SqCLC, had distant metastasis, had lung cancer of an unknown pathological type, had missing data for sex, were aged <20 years, had unclear staging, or exhibited a non-SqCLC histology. In addition, we excluded patients with lung SqCLC if they did not receive surgery for stages I and II cancer, underwent concurrent chemoradiotherapy (CCRT) for stage III cancer after receiving their lung SqCLC diagnosis, received insufficient chemotherapy (concurrent chemotherapy comprising two agents with at least one containing platinum), or did not receive a platinum-based chemotherapy regimen. We also excluded patients who received only sequential chemotherapy and radiation therapy (RT), chemotherapy alone, or RT alone. Standard CCRT comprises concurrent chemotherapy with 2 agents containing platinum and thoracic RT with 6000 cGy administered in daily fractions [18,19,20].

### 2.3. PSM and Covariates

After adjustments were made for confounders, we used a time-dependent Cox proportional hazards model to calculate the time from the index date to all-cause death for patients with both lung SqCLC and mild or moderate-to-severe diabetes. To reduce the effects of potential confounders on the comparison of all-cause death between the mild and moderate-to-severe diabetes groups (which comprised patients with both diabetes and lung SqCLC), the patients included in the current study were propensity-score matched. The variables used for matching were sex, age, AJCC clinical stage, income level, urbanization, Charlson comorbidity index (CCI) score, comorbidities (for example, chronic obstructive pulmonary disease [COPD], chronic bronchitis, emphysema, acute upper respiratory tract infection, asthma, pneumoconiosis, cardiovascular diseases, acute myocardial infarction [AMI], stroke, tuberculosis [TB], obesity), current smoking habit, alcohol-related disease, and diabetes duration (1–1.99, 2–2.99, 3–3.99, 4–4.99, and ≥5 years) (Table 1). Repeated comorbidities were excluded from CCI scores to prevent repetitive adjustments in the multivariate analysis of the current study. Comorbidities were determined in accordance with the *ICD-9-CM* or *ICD-10-CM* codes in the main inpatient diagnosis records of a patient or if a patient made ≥2 outpatient visits within 1 year. Comorbidities that presented within the 6 months preceding the index date were recorded. Continuous variables are presented as means ± standard deviations or medians (first and third quartiles), where appropriate. We matched patients at a ratio of 1:1 by using the greedy method; sex, age, AJCC clinical stage, income level, urbanization, CCI score, comorbidities, current smoking habit, alcohol-related disease, and diabetes duration were propensity-score matched within a caliper of 0.2 [21]. Matching is a common technique for selecting controls with identical background covariates as study participants; investigators perform matching when it is necessary to control for and minimize the differences among study participants. In the current study, multivariate Cox regression analysis was performed to calculate hazard ratios with 95% confidence intervals (CIs) to determine whether the variables listed in Table 1 are the potential independent predictors of all-cause death.

### 2.4. Sensitivity Analysis

**Figure 1:**
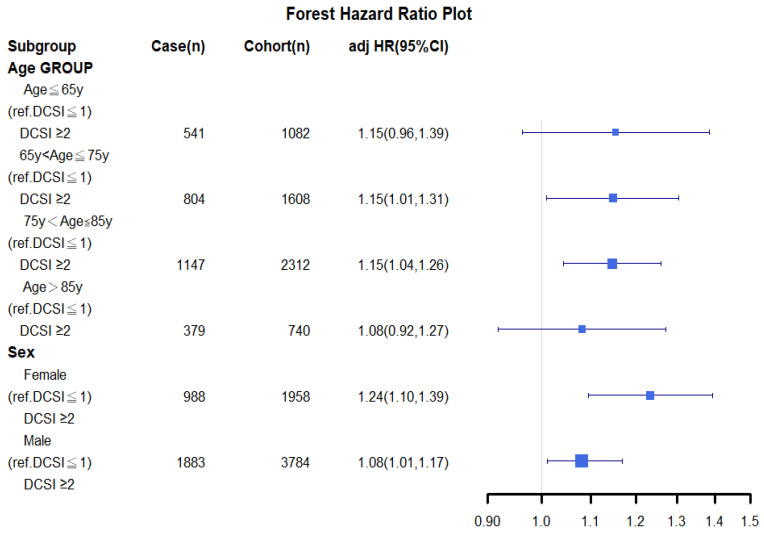
Sensitivity analysis of sex- and age-stratified groups (using inverse probability of treatment weighting) for all-cause death in patients with both lung squamous cell carcinoma and mild or moderate-to-severe diabetes. Abbreviations: aHR, adjusted hazard ratio; y, years; aDCSI, adapted Diabetes Complications Severity Index; CI, confidence interval; ref., reference group.

A sensitivity analysis of various cancer types was conducted using inverse probability of treatment weighting (IPTW) for all-cause death in propensity score–matched mild and moderate-to-severe diabetes groups; it was conducted to clarify the association of mortality with diabetes severity among patients stratified by age, sex, and clinical stage (Figure 1). All of the analyses were adjusted for the covariates in Table 2.

### 2.5. Statistical Analysis

All of the analyses were performed using SAS version 9.4 (SAS Institute, Cary, NC, USA). The matching procedure was implemented using PROC PSMATCH in SAS [22]. In a two-tailed Wald test, *p* < 0.05 was regarded as significant. OS was estimated using the Kaplan–Meier method, and differences between the mild and moderate-to-severe diabetes groups with lung SqCLC were determined by performing a stratified log-rank test and subsequently comparing the survival curves (stratified according to matched sets) [23].

## 3. Results

### 3.1. PSM and Study Cohort

PSM yielded a final cohort of 5742 patients with lung SqCLC (2871 patients in the mild diabetes group [aDCSI = 0–1] and 2871 patients in the moderate-to-severe diabetes group [aDCSI ≥ 2]) who were eligible for further analysis; their characteristics are listed in Table 1. Age distribution was balanced between the two groups (Table 1). Furthermore, after head-to-head PSM was performed, no significant differences in sex, age, AJCC clinical stage, income level, urbanization, CCI score, comorbidities, current smoking habit, alcohol-related disease, and diabetes duration were observed between the two groups. All-cause death, the primary endpoint, significantly differed between the patients with lung SqCLC in the moderate-to-severe diabetes group and the patients with lung SqCLC in the mild diabetes group (*p* < 0.001; Table 1). Due to the high collinearity of diabetes severity, use of diabetic medications and number of diabetic medications taken were unmatched; adjustments were made for these variables in the multivariable Cox model. Table 1 reveals that relative to the patients in the mild diabetes group, the patients in the moderate-to-severe diabetes group took a significantly higher number of diabetic medications and were prescribed diabetic medications at a significantly higher frequency.

### 3.2. Prognostic Factors for All-Cause Death of Lung SqCLC after Multivariate Cox Regression Analysis

The results of a multivariate Cox regression analysis indicated that the patients with lung SqCLC and moderate-to-severe diabetes had a significantly shorter OS (Table 2) relative to the patients with lung SqCLC and mild diabetes. Except for older age (>65 years), male sex, and an aDCSI score of ≥2, no other significant differences were observed for explanatory variables. In the multivariate Cox regression analysis, the adjusted hazard ratio (aHR; 95% CI) of all-cause mortality for the patients with lung SqCLC and mild diabetes relative to the patients with lung SqCLC and moderate-to-severe diabetes was 1.17 (1.08–1.28; *p* = 0.0005). The aHRs (95% CIs) of all-cause mortality for the patients aged 66–75 years, 76–85 years, and >85 years (relative to the patients aged ≤65 years) were 1.33 (1.13–1.57), 2.03 (1.76–2.37), and 3.12 (2.60–3.71), respectively (Table 2). The aHR (95% CI) of all-cause mortality for the male patients with lung SqCLC relative to the female patients with lung SqCLC was 1.19 (1.10–1.34).

### 3.3. Sensitivity Analysis of All-Cause Mortality for Lung SqCLC between Mild and Moderate-to-Severe Diabetes Groups (Stratified by Sex and Age)

A stratified analysis of distinct groups stratified by age and sex on the basis of IPTW was performed, and the results are presented as a forest plot in Figure 1. Among the patients with lung SqCLC and moderate-to-severe diabetes, those aged <65 years, 65–74 years, 75–85 years, and >85 years had aHRs (95% CIs) of 1.15 (0.96–1.39), 1.15 (1.01–1.31), 1.15 (1.04–1.26), and 1.08 (0.92–1.27), respectively, indicating a significantly higher risk of mortality relative to the patients with lung SqCLC and mild diabetes for all age groups (Figure 1). Furthermore, among the patients with lung SqCLC and moderate-to-severe diabetes, female and male patients had aHRs (95% CI) of 1.24 (1.10–1.30) and 1.08 (1.01–1.17), respectively, for all-cause mortality relative to the patients with lung SqCLC and mild diabetes.

### 3.4. Kaplan–Meier Survival Curve of Mild and Moderate-to-Severe Diabetes Groups for of Lung SqCLC

**Figure 2:**
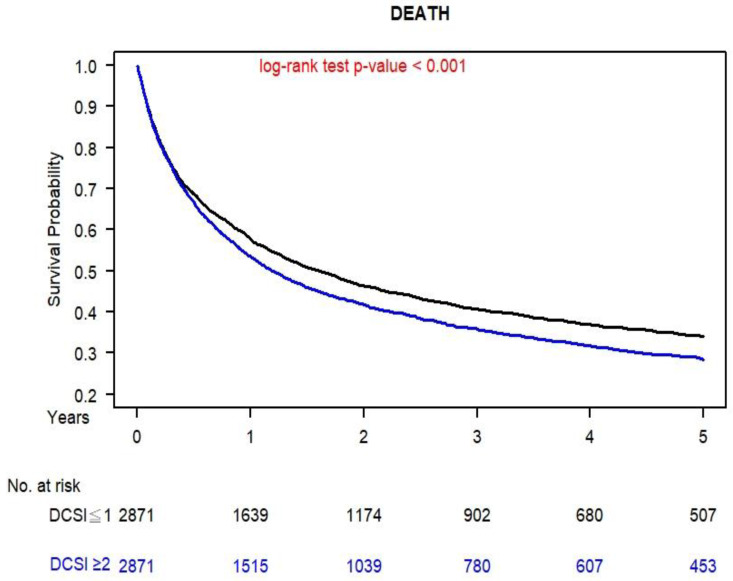
Kaplan–Meier overall survival curves for patients with both lung squamous cell carcinoma and mild or moderate-to-severe diabetes.

Figure 2 presents the OS curves for the propensity score–matched patients with lung SqCLC and mild or moderate-to-severe diabetes; the curves were obtained using the Kaplan–Meier method. The 5-year OS for the patients with moderate-to-severe diabetes and those with mild diabetes were 40.12% and 32.94%, respectively (*p* < 0.001).

## 4. Discussion

Studies have reported that patients with both lung cancer and diabetes have a poorer survival rate than those with lung cancer but without diabetes [11,24,25,26]; this is especially true for women [11]. However, no clear data have been obtained with respect to the clinical stages of lung cancer and the types of lung cancer (SqCLC, adenocarcinoma, small cell carcinoma, and large cell carcinoma) [11,24,25,26]. Survival and treatments administered differ for distinct types of lung cancer [27], especially those involving driver gene mutations such as epidermal growth factor receptor mutation in lung adenocarcinoma [28,29]. Therefore, we focused on lung SqCLC, for which consistent treatments are administered for each stage, and no driver gene mutations are involved. Studies have indicated that diabetes is a poor prognostic factor of OS for lung cancer [11,24,25,26], but no evidence has been produced to demonstrate the association of diabetes severity with lung cancer survival. In our study, we conducted head-to-head PSM to mimic a randomized controlled trial (RCT) and estimate the survival impact of diabetes severity (aDCSI of 0–1 or ≥2) on lung SqCLC. Our large-scale study is the first to demonstrate that moderate-to-severe diabetes (aDCSI ≥ 2) can result in poor OS in patients with lung SqCLC who are receiving standard treatments (regardless of age and sex).

Diabetes can influence lung cancer progression and outcome through several mechanisms, including hyperglycemia, hyperinsulinemia, metabolic dysregulation in cancer cells, and chronic inflammation, all of which are associated with cell proliferation and cancer progression [30,31]. Elevated insulin levels, which represent insulin resistance, can promote cancer through the insulin-like growth factor-1 (IGF-1) pathway [32]. The IGF-1 pathway is regarded as a key promoter of tumor progression [33,34], and IGF-1 receptor inhibitors can contribute to cancer therapy [35,36]. In addition, hyperglycemia and metabolic dysregulation in cancer cells may accelerate the proliferation of lung cancer cells through epidermal growth factor expression, the reversal of the Warburg effect, and the reactivation of oxidative phosphorylation [37,38,39]. Furthermore, patients with both lung cancer and moderate-to-severe diabetes may receive fewer standard treatments for lung cancer relative to patients with only lung cancer because of the greater risk of chemotherapy-related toxicity [40]. At the time of writing, studies on mortality outcomes for individuals with both lung cancer and diabetes have produced conflicting results [41,42,43,44]. These conflicting data may be related to the survival impact of diabetes severity in patients with lung cancer, which can be attributed to the decision-making associated with cancer treatments, tumor response, and poor diabetes control combined with cancer progression [37,38,39,41,42,43,44]. Collectively, the aforementioned findings indicate that moderate-to-severe diabetes is proportionally related to the poor control of hyperglycemia, hyperinsulinemia, or chronic inflammation, which exacerbates metabolic dysregulation in lung cancer and results in poorer OS relative to mild diabetes with lung SqCLC.

Few studies have examined the effects of diabetes therapy on lung cancer outcomes, and those that did were retrospective studies with small sample sizes [11,45,46,47,48]. Studies have produced controversial data suggesting that the use of antidiabetic drugs (particularly metformin) reduces mortality in cancer patients [11,45,46,47,48]. By contrast, several studies have suggested that relative to various therapies (for example, insulin and sulfonylureas), metformin use improves survival outcomes for patients with non–small cell lung carcinoma [46,49]. Other studies have reported that metformin use has no effect or even leads to poorer survival for participants with lung cancer [47,48]. Although antidiabetic drugs were not matched in our study (Table 1), our results are consistent with the findings of the studies in which metformin or other antidiabetic drugs were revealed to have no anticancer effects [11,47,48]. The epidemiological data on the effects of various types of diabetes therapy on lung cancer outcomes are scarce and inconsistent; furthermore, the findings of relevant studies may have been affected by inconsistent or unclear selection of patients with lung cancer, unclear lung cancer histology, unclear clinical stages, and inhomogeneous confounding factors [46,47,48,49]. Our findings, which were obtaining after balancing covariates (Table 1) and eliminating selection bias, reveal that metformin and other antidiabetic drugs are not associated with survival outcomes for lung SqCLC (Table 2).

In the current study, only patients with both diabetes and lung SqCLC were enrolled for further analysis to avoid the influence of various survival effects, inconsistent treatments, and various driver gene mutations related to other types of lung cancer [27,28,29]. Additionally, almost all of the potential confounding factors associated with OS for lung SqCLC were matched (Table 1), with the exception being antidiabetic drug use for diabetes with varying levels of severity. After PSM was performed, all of the covariates were balanced between mild and moderate-to-severe diabetes with lung SqCLC. Evaluating lung SqCLC survival in patients with mild or moderate-to-severe diabetes through an RCT is challenging because lung SqCLC cannot be treated through tangible interventions [50]. Striking a balance among the confounding factors of lung SqCLC survival in patients with mild diabetes (case group) or moderate-to-severe diabetes (control group)—a main design requirement for RCTs—is difficult to achieve [50]. However, PSM can address this problem by maintaining a balance among the confounding factors for the case and control groups. PSM is the recommended strategy for estimating the effects of covariates in studies that may be affected by potential bias [21,51]. Our study is the first to use a PSM-based design to mimic an RCT for evaluating the real-world survival impact on patients with mild or moderate-to-severe diabetes along with lung SqCLC who are receiving standard treatments.

After PSM was performed, the multivariable Cox model did not reveal any significant difference in OS for most covariates between the mild and moderate-to-severe diabetes groups; the notable exceptions were age and sex. Thus, residual imbalances in sex and age may have remained in our population [52,53]. To clarify the effect of diabetes severity and the survival outcomes of lung SqCLC for patients who receive standard treatments, we conducted a sensitivity analysis of all-cause mortality for lung SqCLC and compared the mild and moderate-to-severe diabetes groups, which were stratified by sex and age. The aHRs for mortality were all more than 1 for patients from all age groups and both female and male patients. However, the aHRs of all-cause death for various levels of diabetes severity with lung SqCLC was not significantly different (Figure 1) for the age groups of ≤65 and >85 years because of the small sample sizes of these two age groups; nevertheless, their aHRs were more than 1. The current study is the first to verify that aDCSI is an independent prognostic factor for the OS of patients (regardless of age or sex) with both diabetes and lung SqCLC who are receiving standard treatments (Figure 1 and Figure 2).

Our study is also the first to verify the association of diabetes severity with the OS of patients with lung SqCLC who are receiving standard treatments. An aDCSI of ≥2 is an independent prognostic factor for the OS of patients with both diabetes and lung SqCLC who are receiving standard treatments. Our findings not only revealed that diabetes is a poor prognostic factor for OS in lung SqCLC but also that diabetes severity is an independent prognostic factor for the OS of patients with both diabetes and lung SqCLC who are receiving standard treatments. On the basis of the clinical stage, stratified results that were obtained after PSM, the complications, severity, and poor control of diabetes were revealed to be associated with poor OS in patients with both diabetes and lung SqCLC who were receiving standard treatments. Therefore, patients with diabetes who are also diagnosed with lung SqCLC should maintain excellent diabetes control and prevent their diabetes from progressing to a severe status (aDCSI ≥ 2) to improve their OS. Our results suggest that diabetes prevention medicine is associated with the oncologic outcomes of lung cancer. Our findings can serve as valuable references for endocrinologists, family medicine physicians, and oncologists. Additionally, we further clarified why patients with lung SqCLC can exhibit different survival outcomes even though they have similarly staged cancers and undergo standard treatments; this is a phenomenon that is especially prominent among patients with diabetes of varying severity. The prevention of diabetes progression (prevent aDCSI from progressing to ≥2) is an increasingly crucial public health objective for which health policies must be established. There is no solid data to prove the conceivable oncologic outcomes for lung SqCLC patients with different diabetes severity. Evidence-based data and findings from our study can support the imagines (the association of diabetes severity and mortality with lung SqCLC) to be true in the real-world, instead of conceivable hypothesis. Our study is also the first to verify the association of diabetes severity with the OS of patients with lung SqCLC who are receiving standard treatments. Moreover, the aDCSI score during the date of lung cancer diagnosis and our results hint prevention of diabetes progression is necessary for patients with diabetes; it affects not only diabetes control but also improves survival for patients with lung SqCLC. Clinical studies of lung cancer treatments have indicated that increasing the 5-year OS of patients with lung SqCLC by 8% through an intervention with *p* < 0.0001 is a difficult task [54]. Cancer treatments require substantial medical resources and represent a considerable financial burden for patients and governments [55]. By contrast, the prevention of diabetes progression is a straightforward and cost-effective endeavor that may be associated with an improved OS for patients with lung SqCLC.

The strength of our study is that it is the first large-scale, long-term follow-up, and comparative cohort study to compare the primary endpoints of OS between patients with diabetes who have aDCSI scores of 0–1 and those with aDCSI scores of ≥2. The covariates between the two groups were homogenous for the patients with lung SqCLC, and PSM was performed to eliminate selection bias (Table 1). To date, no study has estimated the survival effect of diabetes severity on all-cause death in patients with lung SqCLC who are receiving standard treatments (surgery for stages I and II and CCRT for stage III). Our study revealed that the poor prognostic factors for OS in patients with both diabetes and lung SqCLC are an aDCSI score of ≥2, male sex, and older age (Table 2); this finding is consistent with those of other cancer studies [56,57]. Among patients with both diabetes and lung SqCLC, those with an aDCSI score of ≥2 have poorer OS than those with an aDCSI of 0–1 (Figure 2). Research on lung SqCLC has been scant and has tended to not distinguish clearly between clinical stages; our study is the first to investigate the survival effects of aDCSI on all-cause death in patients with both diabetes and lung SqCLC (clear stages). Our findings should be considered in future clinical practice and prospective clinical trials to prevent the progression of diabetes to a DCSI level of ≥2; this goal can contribute to improving the OS of patients with both diabetes and lung SqCLC.

The current study has several limitations. First, because all of the enrolled patients with were Asian, their corresponding ethnic susceptibility relative to non-Asians remains unclear; therefore, caution should be taken when extrapolating our results to non-Asian populations. However, no study has reported significant differences in oncological outcomes between Asian and non-Asian survivors of lung SqCLC. Second, the diagnoses of all comorbid conditions were based on *ICD-9-CM* codes. The Taiwan Cancer Registry Administration randomly reviews charts and interviews patients to verify the accuracy of the diagnoses, and hospitals with outlier charges or practices may be audited and heavily penalized if malpractice or discrepancies are identified. Accordingly, to obtain crucial information on population specificity and disease occurrence, a large-scale randomized trial is required to compare carefully selected patients with both diabetes and lung SqCLC who are grouped on the basis of their aDCSI scores (0–1 or ≥2). Finally, the Taiwan Cancer Registry database does not contain information regarding dietary habits or body mass index, which may be risk factors for OS. Despite these limitations, the major strength of the current study is the use of data from a nationwide population-based registry with detailed baseline information. A lifelong follow-up was possible through the linkage of the registry with the national cause-of-death database. Given the magnitude and statistical significance of the observed effects in the current study, the aforementioned limitations are unlikely to have affected our conclusions.

## 5. Conclusions

Severe diabetes (aDCSI ≥ 2) is an independent prognostic factor of OS for patients with both diabetes and lung SqCLC who are receiving standard treatments. Preventing diabetes progression is necessary for patients with diabetes and lung SqCLC because it not only improves their diabetes control but also their OS in relation to lung SqCLC.

## Figures and Tables

**Table 1:** Characteristics of patients with both lung squamous cell carcinoma and mild or moderate-to-severe diabetes (after propensity score matching).

	aDCSI 0–1		aDCSI ≥ 2		
	N = 2871	%	N = 2871		*p*-Value
Sex					
Female	970	33.79%	988	34.41%	0.6163
Male	1901	66.21%	1883	65.59%	
Age	73.18 ± 10.94		74.73 ± 10.32		0.1019
Age group (y)					
Age ≤ 65	541	18.84%	541	18.84%	0.9015
65 < Age ≤ 75	804	28.00%	804	28.00%	
75 < Age ≤ 85	1165	40.58%	1147	39.95%	
Age > 85	361	12.57%	379	13.20%	
AJCC clinical stage					
Stage I	143	4.98%	143	4.98%	1.0000
Stage II	280	9.75%	280	9.75%	
Stage IIIA	725	25.25%	725	25.25%	
Stage IIIB/C	1723	60.01%	1723	60.01%	
Income level (NTD)					
Low-income	41	1.43%	44	1.53%	0.8372
≤10,000	968	33.72%	970	33.79%	
10,001–15,000	718	25.01%	726	25.29%	
15,001–20,000	918	31.97%	914	31.83%	
20,001–30,000	112	3.90%	111	3.87%	
30,001–45,000	68	2.37%	65	2.26%	
>45,000	46	1.60%	41	1.43%	
Urbanization					
Rural	982	34.20%	1014	35.32%	0.3752
Urban	1889	65.80%	1857	64.68%	
CCI Score					
≥1	2341	81.54%	2341	81.54%	1.000
Comorbidities					
COPD	1948	67.85%	1995	69.49%	0.1812
Chronic bronchitis	1541	53.67%	1544	53.78%	0.9284
Emphysema	247	8.60%	232	8.08%	0.9357
Acute upper respiratory tract infection	1243	43.30%	1271	44.27%	0.4564
Asthma	1130	39.36%	1151	40.09%	0.6920
Pneumoconiosis	67	2.33%	54	1.88%	0.2323
Cardiovascular diseases	1537	54.54%	1544	53.78%	0.8205
AMI	208	7.24%	216	7.52%	0.8727
Stroke	324	11.28%	325	11.32%	0.9441
TB	395	13.76%	397	13.83%	0.9047
Obesity	74	2.58%	70	2.44%	0.8407
Current smoking habit	1109	38.63%	1110	38.67%	0.9451
Alcohol-related disease	431	15.01%	434	15.11%	0.7929
Diabetic medication use					
Metformin	1546	53.85%	1682	58.59%	0.0003
Sulfonylurea	1553	54.09%	1714	59.70%	<0.0001
Meglitinide	298	10.38%	355	12.37%	0.0178
α-glucosidase inhibitors	468	16.30%	632	22.01%	<0.0001
Thiazolidinediones	289	10.07%	449	15.64%	<0.0001
Dipeptidyl peptidase-4 inhibitors	226	7.87%	370	12.89%	<0.0001
Glucagon-like peptide-1	201	7.00%	374	13.02%	<0.0001
SGLT2 inhibitors	231	8.05%	402	14.00%	<0.0001
Insulin	482	16.79%	696	24.24%	<0.0001
Number of diabetic medications taken					<0.0001
0	765	26.65%	522	18.18%	
1	478	16.65%	530	18.46%	
2	413	14.39%	352	12.26%	
≥3	1215	42.32%	1467	51.10%	
Diabetes Duration, Years; (Mean ± SD)	4.63 ± 2.15		4.43 ± 2.13		0.8926
1–1.99 year	142	4.95%	148	5.15%	
2–2.99 years	281	9.79%	285	9.93%	
3–3.99 years	724	25.22%	727	25.32%	
4–4.99 years	1001	34.87%	999	34.80%	
≥5 years	723	25.18%	712	24.80%	
Death	1907	66.42%	2035	70.88%	0.0003
Mean follow-up, Year; (Mean ± SD)	2.44 ± 3.24		2.18 ± 2.83		<0.0001
Median follow-up, Year; Median (IQR, Q1, Q2)	1.37 (0.41, 3.87)		1.13 (0.30, 3.77)		0.0019

CCI, Charlson comorbidity index; IQR, interquartile range; SD, standard deviation; NTD, New Taiwan dollars; N, number; y, years; aDCSI, adapted Diabetes Complications Severity Index; N, number; y, years; SGLT2, sodium-glucose cotransporter-2; AMI, acute myocardial infarction; TB, tuberculosis; COPD, chronic obstructive pulmonary disease.

**Table 2:** Multivariable Cox proportional regression analysis of all-cause death among propensity score–matched patients with both lung squamous cell carcinoma and mild or moderate-to-severe diabetes.

	aHR *	95% CI		*p*-Value
aDCSI scores (Ref. aDCSI: 0–1)				
aDCSI ≥2	1.17	1.08	1.28	0.0005
Sex (Ref. female)				
Male	1.19	1.10	1.34	0.0002
Age (y; Ref. ≤ 65)				
65 < Age ≤ 75	1.33	1.13	1.57	0.0004
75 < Age ≤ 85	2.03	1.76	2.37	<0.0001
Age > 85	3.12	2.60	3.71	<0.0001
AJCC clinical stage (Ref. Stage I)				
Stage II	1.01	0.60	1.04	0.3644
Stage IIIA	1.11	0.89	1.36	0.2262
Stage IIIB/C	1.17	0.66	1.97	0.2120
Income level, NTD (Ref. low income)				
≤10,000	0.87	0.65	1.20	0.4762
10,001–15,000	0.85	0.63	1.20	0.4159
15,001–20,000	0.81	0.59	1.12	0.1876
20,001–30,000	0.71	0.44	1.05	0.1291
30,001–45,000	0.62	0.46	1.03	0.0589
>45,000	0.45	0.25	1.04	0.0598
Urbanization (Ref. rural)				
Urban	0.97	0.87	1.09	0.7351
CCI Scores (Ref. CCI = 0)				
CCI ≥ 1	1.01	0.89	1.15	0.9212
Comorbidities				
COPD (Ref. No)	0.96	0.85	1.05	0.1932
Chronic bronchitis (Ref. No)	0.94	0.86	1.03	0.1153
Emphysema (Ref. No)	1.01	0.92	1.09	0.9301
Acute upper respiratory tract infection (Ref. No)	1.17	0.87	1.66	0.2404
Asthma (Ref. No)	1.04	0.81	1.31	0.9156
Pneumoconiosis (Ref. No)	1.00	0.87	1.12	0.8635
Cardiovascular diseases (Ref. No)	1.20	0.89	1.68	0.2441
AMI (Ref. No)	1.15	0.86	1.40	0.3830
Stroke (Ref. No)	1.02	0.76	1.20	0.8721
TB (Ref. No)	1.04	0.80	1.14	0.5311
Obesity (Ref. No)	1.11	0.80	1.51	0.3420
Current Smoking (Ref. No)	1.20	0.94	1.50	0.2261
Alcohol-related disease (Ref. No)	1.25	0.90	1.51	0.3313
Diabetic medication use				
Metformin	0.82	0.66	1.08	0.1282
Sulfonylurea	0.99	0.70	1.15	0.6544
Meglitinide	0.97	0.89	1.10	0.6761
α-glucosidase inhibitors	1.02	0.91	1.17	0.4553
Thiazolidinediones	1.01	0.82	1.20	0.9241
Dipeptidyl peptidase-4 inhibitors	1.03	0.95	1.28	0.1029
Glucagon-like peptide-1	0.95	0.90	1.04	0.1382
SGLT2 inhibitors	0.97	0.90	1.03	0.1764
Insulin	1.02	0.94	1.06	0.7253
Number of diabetic medications taken (Ref. No antidiabetic drug)				
1	1.14	0.81	1.29	0.2352
2	1.32	0.87	1.57	0.3486
≥3	1.23	0.90	1.43	0.3527
Diabetes Duration (Ref. 1–1.99 years)				
2–2.99 years	1.01	0.92	1.32	0.2932
3–3.99 years	1.04	0.88	1.09	0.6948
4–4.99 years	1.08	0.90	1.16	0.2537
≥5 years	1.09	0.81	1.21	0.9216

aHR, adjusted hazard ratio; CCI, Charlson comorbidity index; NTD, New Taiwan dollars; y, years; aDCSI, adapted Diabetes Complications Severity Index; CI, confidence interval; HR, hazard ratio; ref., reference group; N, number; y, years; SGLT2, sodium-glucose cotransporter-2; AMI, acute myocardial infarction; TB, tuberculosis; COPD, chronic obstructive pulmonary disease. * All covariates presented in Table 2 were adjusted.

## Data Availability

The datasets supporting the study conclusions are included within this manuscript and its additional files.

## Supplementary Materials

The following supporting information can be downloaded at: https://www.mdpi.com/article/10.3390/cancers14102553/s1, Table S1: Adapted Diabetes Complications Severity Index.

## Abbreviations

aHR	adjusted hazard ratio
CI	confidence interval
RCT	randomized controlled trial
PSM	propensity score matching
*ICD-9-CM*	International Classification of Diseases, Ninth Revision, Clinical Modification
*ICD-10-CM*	International Classification of Diseases, Tenth Revision, Clinical Modification
OS	overall survival
CCI	Charlson comorbidity index
IPTW	inverse probability of treatment weighting
NTD	New Taiwan dollars
y	years old
aDCSI	adapted diabetes complications severity index
HR	hazard ratio
AMI	acute myocardial infarction
TB	tuberculosis
COPD	chronic obstructive pulmonary disease
SqCLC	squamous cell carcinoma
AJCC	American Joint Committee on Cancer
CCRT	concurrent chemoradiotherapy
RT	radiation therapy
IGF-1	insulin-like growth factor-1
NSCLC	non–small cell lung carcinoma
