# Supplementary material for: Association of Diabetes Severity and Mortality with Lung Squamous Cell Carcinoma
Source: Cancers (Basel). 2022 May 22;14(10):2553. doi: 10.3390/cancers14102553 (PMC9139965; doi:10.3390/cancers14102553)
Supplement: Supplementary file 1 [file cancers-14-02553-s001.zip › cancers-1732007-supplementary.pdf]

Supplemental Table S1. Adapted Diabetes Complications Severity Index

	No abnormality = 0	Some abnormality = 1	Severe abnormality=2
**Retinopathy**	0	1	2
**Nephropathy**	0	1	2
**Neuropathy**	0	1	
**Cerebrovascular**	0	1	2
**Cardiovascular**	0	1	2
**Peripheral vascular disease**	0	1	2
**Metabolic**	0	1	2

aDCSI scores range 0-13
